# Assessing ChatGPT responses to common patient questions regarding total ankle arthroplasty

**DOI:** 10.1002/jeo2.70138

**Published:** 2024-12-31

**Authors:** Elena Artioli, Francesca Veronesi, Antonio Mazzotti, Silvia Brogini, Simone Ottavio Zielli, Gianluca Giavaresi, Cesare Faldini

**Affiliations:** ^1^ 1st Orthopaedics and Traumatologic Clinic, IRCCS Istituto Ortopedico Rizzoli Bologna Italy; ^2^ Department of Biomedical and Neuromotor Sciences (DIBINEM) Alma Mater Studiorum University of Bologna Bologna Italy; ^3^ Surgical Sciences and Technologies, IRCCS Istituto Ortopedico Rizzoli Bologna Italy

**Keywords:** artificial intelligence, ChatGPT, total ankle arthroplasty

## Abstract

**Purpose:**

Artificial Intelligence is becoming increasingly integrated into healthcare, making it essential to assess its potential as a reliable information source for patient queries in the ambit of orthopaedic surgery. In literature, it is being employed in foot and ankle surgery and total hip arthroplasty. The aim of the present study was to evaluate the ability of Chat Generative Pretrained Transformer (ChatGPT) version 3.5 to give accurate, complete and comprehensive responses to the most common questions which are usually asked by the patient to the surgeon regarding total ankle arthroplasty.

**Methods:**

Ten most common questions were selected by two ankle surgeons and then ChatGPT was used to answer these questions. The responses were analyzed using an accuracy score and the modified DISCERN score to assess clarity. WordCalc software package (educational‐level indices) was used to assess the readability of the responses.

**Results:**

Most of ChatGPT's responses were considered excellent not requiring clarification or satisfactory requiring minimal clarification. Indeed, the accuracy score was 2, suggesting that the overall responses were satisfactory requiring minimal clarification, and DISCERN score mean was 51, which is considered good‐fair.

**Conclusions:**

ChatGPT demonstrates potential as a tool for responding to common patient questions related to total ankle arthroplasty, offering clear and mostly accurate information. While its current performance is based on the available literature, ongoing advancements in artificial intelligence may further enhance its utility in healthcare communication. However, further studies are required to evaluate its role more precisely in patient information and clinical settings.

**Levels of Evidence:**

Not applicable.

**Abbreviations**
AI: artificial intelligence
IRR: interrater reliability
TAA: total ankle arthroplasty

## INTRODUCTION

Total Ankle Arthroplasty (TAA) represents an increasingly favoured and well‐established treatment option for end‐stage ankle osteoarthritis [10].

TAA has been proposed as an alternative to ankle fusion, in an effort to preserve range of motion thus improving functional outcomes [8]. Thanks to the favourable long‐term results and the evolutions in implant materials and design, surgical techniques and surgeon training, the number of TAA is growing rapidly [12, 13]. In the United States, there was a 564% increase in TAA procedures from 2005 to 2017 with a further projected increase of 110%–796% by 2030 [30].

The interest in TAA has surged, captivating not only surgeons but also patients who increasingly seek to preserve optimal ankle functionality. This situation leads patients themselves to inquire with interest about the procedure.

Among the most used means of information are internet search engines and, more recently, artificial intelligence (AI). AI is becoming increasingly integrated into healthcare, making it essential to assess its potential as a reliable information source for patient queries also in the ambit of orthopaedic surgery. AI is employed by an online chatbot that responds to the doubts and questions of users, the Chat Generative Pretrained Transformer (ChatGPT) (OpenAI), who generates a language model tool with the aim of making conversation between public and AI network using a text‐based platform [25]. From November 2022, ChatGPT briefly gained popularity with nearly 100 million users in 2023 [21], showing unlimited potential in various fields of study and giving comprehensive and clear answers to all the queries, and also to health‐related questions [23, 27]. Given its linguistic ability and versatility, the arguments regarding medicine fields have grown rapidly, and also in clarifying orthopaedic doubts [3, 21].

Since ChatGPT has been employed more than other internet research, it is important that the quality of medical information is verified, in order to avoid misinformation.

The aim of the present study was to evaluate the quality of the answers given by ChatGPT to the questions that usually patients ask their surgeon when they undergo TAA. The generated answers were scored by orthopaedic foot and ankle surgeons and by literature on the argument.

## METHODS

### Question selection

After analyzing the frequently asked questions regarding TAA from the websites of the world's top three orthopaedic hospitals, as per the Newsweek ranking [https://www.newsweek.com/rankings/worlds-best-specialized-hospitals-2024/orthopedics], and drawing upon the clinical expertise of two orthopaedic surgeons specialized in foot and ankle surgery, a list of the 10 most prevalent inquiries concerning TAA was curated by two authors (F. V. and E. A.). In cases of disagreement, a consensus was reached between the authors. Then, the 10 questions, listed in Table 1, were asked to the AI platform ChatGPT version 3.5 (https://chat.openai.com/chat) on 28 March 2024.

**Table 1:** Lists of questions and respective ChatGPT responses.

Questions	ChatGPT responses
**When should I get TAA?**	Deciding to get a TAA is a significant medical decision should be made in consultation with your orthopaedic surgeon. TAA is typically considered when conservative treatments, such as medication, physical therapy or bracing, no longer provide relief from ankle pain caused by conditions like severe arthritis or deformities. Here are some factors to consider: Severity of Symptoms: If your ankle pain significantly affects your daily activities and quality of life, despite trying conservative treatments, it may be time to consider surgery. Extent of Damage: TAA is usually recommended for people with advanced arthritis or significant damage to the ankle joint. Your surgeon will assess the extent of joint damage through imaging tests like c‐rays or MRI scans. Age and Activity Level: While TAA can provide pain relief and improved function, it's essential to consider your age and activity level. Younger, more active individuals may have different surgical options or may need to consider the longevity of the replacement joint. Overall Health: Your overall health and medical history plays a crucial role in determining if you're a suitable candidate for surgery. Conditions such as diabetes, obesity or vascular problems may increase the risks associated with surgery. Expectations: Discuss your expectations and goals with your surgeon. TAA can relieve pain and improve mobility, but it may not restore the same level of function as a healthy ankle joint. Understanding the potential benefits and limitations of surgery is essential. Consultation with Specialists: Seek opinions from orthopaedic surgeons who specialize in TAA. They can provide personalized recommendations based on your specific condition and needs. Ultimately, the decision to undergo TAA should be made collaboratively between you and your healthcare team after careful consideration of all relevant factors.
**What are the risks of TAA?**	TAA is a surgical procedure that carries certain risks, as with any major surgery. While many people experience successful outcomes with TAA, it's essential to be aware of potential complications. Some of the risks associated with TAA include: Infection: Infection is a risk with any surgical procedure. Your surgeon will take precautions to minimize this risk, such as administering antibiotics before and after surgery and maintaining sterile conditions in the operating room. However, infections can still occur and may require additional treatment, including antibiotics or, in severe cases, surgical intervention to remove infected tissue or hardware. Blood Clots: Blood clots (deep vein thrombosis) can develop in the legs after surgery, particularly if you're immobile for an extended period. These clots can be dangerous if they travel to the lungs (pulmonary embolism). Your medical team will implement measures to prevent blood clots, such as compression stockings, blood thinners and early mobilization. Implant Failure: While TAA implants are designed to be durable, there's a risk of implant failure over time. This can occur due to factors such as wear and tear, loosening of the components or fracture of the implant. Revision surgery may be necessary if the implant fails. Nerve Damage: Surgery carries a risk of nerve damage, which can result in numbness, tingling, weakness or altered sensation in the foot and ankle. Most nerve injuries resolve over time, but in some cases, they may be permanent. Bone Fracture: Fractures of the bones around the ankle joint can occur during or after surgery. Your surgeon will take precautions to minimize this risk, but fractures may still occur, particularly in individuals with weak or osteoporotic bones. Stiffness and Reduced ROM: While TAA can improve mobility and function, some people may experience stiffness or reduced ROM in the ankle joint following surgery. Physical therapy and rehabilitation can help minimize these effects. Allergic Reactions: Some individuals may have allergic reactions to the materials used in TAA implants. Your surgeon will discuss any known allergies you have and choose an implant material that is suitable for you. It's important to discuss these risks with your surgeon and weigh them against the potential benefits of TAA. Your surgeon will provide personalized recommendations based on your individual health status and treatment goals.
**How common is infection after TAA?**	The incidence of infection after TAA is relatively low but can vary depending on several factors, including the patient's overall health, the surgical technique used and postoperative care protocols. Generally, infection rates for TAA range from 1% to 5%.Orthopaedic surgeons take numerous precautions to minimize the risk of infection during and after surgery. These precautions may include: Preoperative antibiotics: Administering antibiotics before surgery helps reduce the risk of infection. Sterile surgical environment: Surgeons operate in a sterile environment and take precautions to prevent contamination. Postoperative antibiotics: Patients may receive antibiotics after surgery to further reduce the risk of infection. Wound care: Proper wound care and monitoring are crucial during the postoperative period to detect any signs of infection early. Patient education: Patients are educated about the signs and symptoms of infection and instructed on how to care for their surgical incision. Despite these measures, infections can still occur. Certain factors may increase the risk of infection after TAA, including: Diabetes or other medical conditions that weaken the immune system Smoking Obesity Previous infections or surgeries in the same area Poor wound healing If an infection occurs after TAA, prompt treatment is essential to prevent complications. Treatment may involve antibiotics, surgical debridement (removal of infected tissue) or, in severe cases, removal of the implant. While infection is a potential complication of TAA, the overall success rate of the procedure is high, and most patients experience significant improvement in pain and function following surgery. It's essential for patients to communicate with their healthcare providers and follow postoperative instructions to minimize the risk of complications.
**What is the success rate of TAA?**	The success rate of TAA is generally high, with many patients experiencing significant improvement in pain relief and function. However, success rates can vary depending on several factors, including the patient's age, overall health, the underlying condition requiring surgery and the surgeon's experience. Overall, the success rate of TAA is estimated to be around 80%– 90% at 10 years postsurgery. This means that the majority of patients who undergo TAA experience long‐term relief from pain and improvement in mobility and function. Some studies have reported even higher success rates in specific patient populations. Factors that contribute to a successful outcome after TAA include: Patient Selection: Patients who are carefully selected based on factors such as age, activity level and the severity of their ankle condition are more likely to have a successful outcome. Surgeon Experience: The experience and skill of the surgeon performing the procedure play a significant role in the success of TAA. Surgeons who specialize in foot and ankle surgery and have extensive experience with TAA procedures tend to achieve better outcomes. Postoperative Rehabilitation: Following surgery, adherence to a structured rehabilitation programme is crucial for optimizing outcomes. Physical therapy helps improve strength, flexibility and ROM in the ankle joint, facilitating a smoother recovery and better functional outcomes. Patient Compliance: Patient compliance with postoperative instructions, including weight‐bearing restrictions, activity modifications and follow‐up appointments, also influences the success of ankle replacement surgery. Management of Complications: Prompt identification and management of any complications that arise after surgery, such as infection or implant loosening, are essential for achieving a successful outcome. While TAA offers significant benefits for many patients, it is essential to have realistic expectations and understand that no surgical procedure is without risks. Patients should discuss the potential risks and benefits of TAA with their healthcare provider and make an informed decision based on their individual circumstances.
**How long will I be able to drive after TAA?**	The timeframe for when you can resume driving after TAA can vary depending on several factors, including the specific details of your surgery, your overall health and your ability to safely control a vehicle. Generally, it is recommended to wait until you have sufficiently recovered and are no longer taking pain medications that could impair your driving abilities. Here are some general guidelines: Follow Surgeon's Instructions: Your surgeon will provide specific guidance on when it's safe for you to resume driving after TAA. They will consider factors such as the type of anaesthesia used, the extent of your surgery and your individual recovery progress. Wait Until You're Off Pain Medication: If you're taking prescription pain medications, such as opioids, it's important to wait until you no longer need these medications and are not experiencing significant pain that could affect your ability to drive safely. Regain Mobility and Strength: You should be able to operate the pedals and controls of a vehicle comfortably and safely before resuming driving. This may require regaining strength and mobility in your ankle joint, as well as improving your overall balance and coordination through physical therapy and rehabilitation. Consider the Type of Vehicle: If you drive a manual transmission vehicle (stick shift), you may need to wait longer before resuming driving compared to driving an automatic transmission vehicle, as manual transmission cars require the use of both feet. Consult with Your Healthcare Provider: Before you resume driving, it is a good idea to discuss your plans with your surgeon or primary care provider. They can assess your progress, evaluate any lingering mobility issues and provide personalized recommendations based on your individual circumstances. In general, most people are able to resume driving within a few weeks to a couple of months after TAA, but it ultimately depends on how well you're recovering and when you feel confident and capable of safely operating a vehicle. Be sure to prioritize your safety and follow any restrictions or recommendations provided by your healthcare team.
**What physical limitations will I have after TAA? Can I do any activity I want?**	After TAA, while many patients experience significant improvement in pain relief and mobility, there are still some limitations and considerations to keep in mind. While you may be able to resume many activities you enjoy, it's important to avoid high‐impact or high‐stress activities that could put excessive strain on the replaced ankle joint. Here are some general guidelines regarding limitations and activities after TAA: Low‐Impact Activities: Following TAA, you should focus on low‐impact activities that are gentle on the joint. This includes activities such as walking, swimming, cycling and low‐impact aerobic exercises. These activities help improve strength, flexibility and cardiovascular health without placing excessive stress on the replaced joint. Avoid High‐Impact Activities: Activities that involve repetitive pounding or high impact, such as running, jumping or high‐intensity sports like basketball or soccer, should generally be avoided after TAA. These activities can increase the risk of implant wear, loosening or fracture and may compromise the longevity of the replaced joint. Consult with Your Surgeon: Your surgeon will provide specific guidance on activity limitations based on your individual circumstances, including the type of implant used, your age and your overall health. It's important to follow their recommendations closely to ensure the best possible outcome. Gradual Return to Activities: It's essential to gradually reintroduce activities into your routine after TAA, starting with gentle exercises and gradually increasing intensity as you regain strength and mobility. Physical therapy and rehabilitation can help guide you through this process and ensure a safe and effective recovery. Listen to Your Body: Pay attention to how your ankle feels during and after activities. If you experience pain, swelling or discomfort, it may be a sign that you're overdoing it or engaging in activities that are too strenuous for your replaced joint. Listen to your body and adjust your activity level accordingly. While TAA can significantly improve quality of life for many people with debilitating ankle pain, it is important to be mindful of the limitations and to prioritize the long‐term health and function of the replaced joint. By following your surgeon's recommendations and adopting a balanced approach to physical activity, you can maximize the benefits of TAA while minimizing the risk of complications.
**How painful is TAA?**	TAA is a major surgical procedure, and like any surgery, it can cause discomfort and pain during the recovery process. However, advances in surgical techniques, anaesthesia and pain management have helped minimize pain and discomfort associated with TAA. During TAA, you will receive anaesthesia to ensure that you are comfortable and pain‐free throughout the procedure. After surgery, you can expect some level of discomfort as the anaesthesia wears off and the initial postoperative pain sets in. The level of pain experienced can vary from person to person and depends on factors such as individual pain tolerance, the extent of the surgery and any underlying medical conditions. Your healthcare team will provide pain management strategies to help alleviate discomfort and manage pain during the recovery period. This may include a combination of pain medications, such as opioids, NSAIDs and nerve blocks. Additionally, techniques such as ice therapy, elevation and compression may be used to reduce swelling and discomfort in the surgical area. It is important to communicate openly with your healthcare providers about your pain levels and any concerns you may have. They can adjust your pain management plan as needed to ensure that you are as comfortable as possible during the recovery process. While some discomfort and pain are to be expected after TAA, it typically improves over time as you progress through the recovery process. Following your surgeon's postoperative instructions, including rest, elevation and physical therapy, can help minimize pain and promote healing, ultimately leading to a successful outcome from TAA.
**When is AF a better option than TAA?**	AF and TAA are both surgical options for addressing severe ankle arthritis or other conditions causing significant pain and limited mobility. The choice between the two depends on various factors including the patient's age, activity level, overall health, severity of arthritis and personal preferences. Here are some situations where AF might be a better option: Severe Arthritis: AF may be preferred when the arthritis is advanced and involves multiple areas of the joint, making it difficult to achieve satisfactory results with TAA. Younger Patients: AF might be more suitable for younger, active patients who have higher physical demands and may put more stress on an artificial joint. Fusion provides stability but limits motion, which may be acceptable for those who engage in high‐impact activities. Complex Deformities: In cases where there are significant deformities or bone loss around the ankle joint, fusion may be more feasible and reliable compared to TAA. Failed Previous Surgeries: If a patient has undergone multiple failed ankle surgeries or has complications from previous procedures, AF might be the preferred option to address the issues more definitively. Infection Risk: Patients with a high risk of infection or those who have had previous infections in the ankle region may be better suited for fusion, as it eliminates the need for a prosthesis that could be susceptible to infection. However, AF does have drawbacks, primarily the loss of motion in the ankle joint, which can lead to altered walking mechanics and increased stress on adjacent joints. TAA, on the other hand, aims to preserve motion and mimic the natural joint more closely, but it may have a higher risk of complications and may not be suitable for all patients. Ultimately, the decision between AF and TAA should be made in consultation with an orthopaedic surgeon who can evaluate the individual patient's condition and preferences to determine the most appropriate treatment option.
**How long will I stay in hospital for TAA?**	The length of hospital stay for TAA can vary depending on several factors, including the specific surgical technique used, your overall health and how well you recover from surgery. In general, the typical hospital stay for TAA ranges from one to three days.Here is what you can generally expect regarding the hospital stay for TAA: Day of Surgery: On the day of surgery, you will typically arrive at the hospital or surgical centre several hours before the scheduled procedure. After the surgery, you will be taken to a recovery area where medical staff will monitor your condition as you wake up from anaesthesia. Once you are stable, you will be transferred to your hospital room. First Day Postsurgery: The first day after TAA is focused on pain management, monitoring for any signs of complications and starting physical therapy. You will work with physical therapists to begin gentle exercises to promote circulation and prevent stiffness in your ankle joint. Subsequent Days: Depending on your progress and how well you are able to tolerate weight‐bearing activities, you may stay in the hospital for another day or two. During this time, you will continue to receive pain management, antibiotics (if prescribed) and physical therapy. The goal is to ensure that you are safe and comfortable before being discharged home. Discharge Planning: Before you are discharged from the hospital, your healthcare team will provide instructions for postoperative care, including wound care, medication management, activity restrictions and follow‐up appointments. They may also arrange for any necessary medical equipment or home care services to assist with your recovery. It is essential to follow your healthcare provider's instructions carefully and attend all scheduled follow‐up appointments to monitor your progress and address any concerns. Additionally, be sure to have a plan in place for support at home during the initial stages of your recovery, as you may need assistance with daily activities while you regain strength and mobility in your ankle.
**What is the postoperative ROM of the ankle after TAA?**	The postoperative ROM of the ankle after TAA can vary depending on several factors, including the specific surgical technique used, the condition of the surrounding soft tissues and the patient's individual anatomy and rehabilitation progress. Generally, the goal of TAA is to restore as much functional ROM as possible while maintaining stability and joint alignment. Following TAA, patients typically undergo a period of rehabilitation aimed at restoring flexibility, strength and mobility in the ankle joint. Physical therapy plays a crucial role in this process, and therapists work with patients to gradually increase ankle mobility through a series of exercises and stretches. While the exact postoperative ROM can vary from person to person, studies have shown that many patients achieve satisfactory ankle motion following TAR surgery. Research indicates that the average ROM after TAA ranges from approximately 20°–30° of dorsiflexion (bending the foot upward) and 30°–40°degrees of plantar flexion (pointing the foot downward). These ranges of motion are generally sufficient for most activities of daily living. It is important to note that achieving maximal ROM after TAR may take time, and some patients may experience stiffness or limitations in ankle mobility, particularly in the early stages of recovery. However, with diligent rehabilitation and adherence to postoperative protocols, many patients are able to achieve functional ROM and return to activities they enjoy. It is essential for patients to work closely with their healthcare providers, including orthopaedic surgeons and physical therapists, to monitor progress, address any concerns and develop an individualized rehabilitation plan tailored to their specific needs and goals. By following postoperative instructions and participating actively in rehabilitation, patients can optimize their outcomes and regain as much ankle motion as possible after TAR surgery.

Abbreviations: AF, ankle fusion; MAR, magnetic resonance imaging; NSAID, nonsteroidal anti‐inflammatory drug; ROM, range of motion; TAA, total ankle arthroplasty; TAR, total ankle replacement.

The search yielded 10 answers total, 1 per question (*n *= 10). ChatGPT responses were recorded after the initial query, and no follow‐up or repeat queries were conducted (Table 1).

### ChatGPT response analysis

Two separate scoring systems were used to evaluate the quality of the responses generated by ChatGPT: the modified DISCERN [7], and Mika et al. response accuracy score [21].

DISCERN is a well validated and used tool employed from 1990s and composed by 16 questions: DISCERN 1, composed by eight questions that evaluate the reliability of the publication and DISCERN 2, composed by seven questions that determine the quality of the source base of the authors. Finally, DISCERN 3 is the total score and unites quality and source information. It was originally employed for written medical information, and now it is largely used to assess ChatGPT questions. The final score is divided into EXCELLENT (63–75 points), GOOD (51–62 points), FAIR (39–50 points), POOR (27–38 points) and VERY POOR (16–26 points) (Table 2) [16].

**Table 2:** Mika et al. [21] response accuracy score and DISCERN score items with respective values.

Mika et al. response accuracy score		DISCERN score	
**1**	Excellent response not requiring clarification	**63–75**	Excellent
**2**	Satisfactory requiring minimal clarification	**51–62**	Good
**3**	Satisfactory requiring moderate clarification	**39–50**	Fair
**4**	Unsatisfactory requiring substantial clarification	**28–38**	Poor
		**16–27**	Very poor

The Mika et al. response accuracy score is used for accuracy with an evidence‐based approach: (1) ‘excellent response not requiring clarification’, (2) ‘satisfactory requiring minimal clarification’, (3) ‘satisfactory requiring moderate clarification’ or (4) ‘unsatisfactory requiring substantial clarification’ (Table 2) [21].

The responses of ChatGPT were scored by two authors (F. V. and E. A.) of our orthopaedic research team.

Finally, the reading level required to understand ChatGPT's responses was calculated using the publicly available WordCalc software package (https://www.wordcalc.com/readability/). Each response's content was pasted as plaintext into the platform for analysis. The following formulas were used to assess readability: (1) Flesch‐Kincaid grade level; (2) Gunning Fog Index; (3) Coleman‐Liau index; (4) Simple Measure of Gobbledygook Index; (5) automated readability index. All five formulas report readability in the number of years of education (grade level) required to properly understand a text. These tests were chosen because they have been routinely used in prior literature assessing health education material readability [15].

Interrater reliability (IRR) was assessed with intraclass correlation coefficients and it is the level of agreement between raters or judges. It was calculated as follows: IRR = TA/(TR∗R)∗100 where TA is Total Number of Agreements, TR is Total Number of Raters and R is Total Number of Scores Given by Each Rater.

This study did not require institutional review board approval because no patients or protected information was involved in this study.

## RESULTS

The list of questions asked to ChatGPT and the corresponding ChatGPT‐generated responses to those questions are listed in Table 1. The Mika et al. response accuracy and DISCERN scores for each response are also provided in Table 3 [21].

**Table 3:** Mika et al. response accuracy score and mean DISCERN score for each ChatCPT responses [21].

Questions	Mika et al. response accuracy score (Author 1)	Mika et al. response accuracy score (Author 2)	Mean DISCERN Score (Author 1)	Mean DISCERN Score (Author 2)
**When should I get TAA?**	1	2	55 (Good)	53 (Good)
**What are the risks of TAA?**	2	2	48 (Fair)	40 (Fair)
**How common is infection after TAA?**	2	2	48 (Fair)	50 (Fair)
**What is the success rate of TAA?**	2	2	55 (Good)	51 (Good)
**How long will I be able to drive after TAA?**	1	1	55 (Good)	59 (Good)
**What physical limitations will I have after TAA? Can I do any activity I want?**	1	1	55 (Good)	62 (Good)
**How painful is TAA?**	3	3	45 (Fair)	39 (Fair)
**When is AF a better option than TAA?**	2	2	55 (Good)	51 (Good)
**How long will I stay in hospital for TAA?**	4	4	45 (Fair)	39 (Fair)
**What is the postoperative range of motion of the ankle after TAA?**	1	1	55 (Good)	62 (Good)

Abbreviations: AF, ankle fusion; TAA, total ankle arthroplasty.

### ChatGPT response analysis

#### ChatGPT response analysis of question 1 (excellent response not requiring clarification)

The ChatGPT response is in accordance with literature and surgeon experience that promote TAA after nonsurgical treatments fail. Degenerative pathologies of the ankle joint, such as osteoarthritis may cause substantial pain and functional limitations, which negatively affects patients' health‐related quality of life [33]. The most common cause of the prevalence of symptomatic radiographic osteoarthritis of the ankle (3.4% of ankle diseases) is a previous joint trauma [11].

Nonsurgical treatments, prevalently employed in early osteoarthritis, are represented by activity modifications, weight loss, orthotic bracing and corticosteroid injection, while surgical ones include AF and TAA, in case of end‐stage osteoarthritis [37].

TAA was first introduced in the 1970s as an alternative to ankle fusion and has emerged as a viable solution, aiming to restore joint function and reduce pain. The improvements in new generation implants lead to a rise in TAA [31].

#### ChatGPT response analysis of question 2 (satisfactory response requiring minimal clarification)

ChatGPT gives a satisfactory response because takes into consideration several risks associated with TAA and other surgery. However, the percentage of failure is not detailed. In literature, early failure/revision rates for TAA have a mean of 9.8% (1.3%–32.3%) after 5 years of follow‐up [27]. Despite improvements in implant design and reductions in overall failure rates, a wide range of other complications, often sporadically are reported in the literature. The overall postoperative complications are (1) intraoperative fracture; (2) postoperative fracture; (3) malpositioning/technical error; (4) wound‐healing problems; (5) superficial infection; (6) Periprosthetic Joint Infection; (7) aseptic loosening/osteolysis; (8) implant‐related complications (polyethylene fracture or dislocation, edge loading); (9) chronic pain (due to stiffness or heterotopic ossification); (10) soft‐tissue injuries (nerves or tendons); (11) subsidence; (12) thromboembolism; (13) Complex Regional Pain Syndrome; (14) amputation [15].

#### ChatGPT response analysis of question 3 (satisfactory response requiring minimal clarification)

In ChatGPT response seems that the percentage of infection after TAA is lower than reality. Indeed, among the complications of TAA, Periprosthetic Joint Infection is a high‐grade one, with a rate between 0%–13%, leading to the failure of the implant, difficult resolution of the infective process and complex revision surgery [23]. In addition, several risk factors are detailed in ChatGPT response, but not all. Some are the identified risk factors, such as the low body mass index, inflammatory arthritis, peripheral vascular disease, diabetes, chronic lung disease, hypothyroidism, low preoperative AOFAS hindfoot scores, prior ankle surgery and wound healing problem more than 14 days postoperatively [4, 19].

#### ChatGPT response analysis of question 4 (excellent response not requiring clarification)

ChatGPT response is appropriate because it is in accordance with literature data and the percentages of TAA success are in accordance.

Thanks to the current improvements in the design of the third‐generation implants, TAA shows a long‐term survivorship rate (nearly 85% at 10 years) [25] and the reported complication rate is low [13]. In addition, factors that contribute to a successful outcome are in line with those in the literature. The success of a TAA is principally based on (1) surgeon factor: surgeons with less or lack of experience for complications management have more intraoperative complications; (2) Prosthetic factor: for example, polyethylene gives more implant instability and subsequent severe particulate wear than the metal components. In addition, anatomic conical shape of the talar component reduces adjacent joint degeneration; (3) intraoperative procedures: minimal bone resection reduces postoperative fractures; (4) patient related factors: patients with young age, high activity level and low severity of ankle condition are more likely to have a successful surgery [39].

#### ChatGPT response analysis of question 5 (excellent response not requiring clarification)

ChatGPT response is well supported by data in literature and data of the guidelines drawn up by surgeons, although the timing may vary from patient to patient.

It is important to evaluate the safe return to driving after TAA, because it is more significant than the safe return to driving after other lower‐extremity joint surgery. Indeed, ankle is considered the most important mover in braking motions over the knee and hip [32]. Some studies have suggested that most patients can safely return to drive at 9 weeks after open reduction and internal fixation of the right ankle and nearly 90% of patients achieve a safe brake‐reaction time within 6 weeks from TAA. Surgeons might counsel patients with persistent pain and stiffness at 6 weeks to delay driving for an additional 3 weeks, until 9 weeks [29]. However, there are differences in range of motion after TAA that make a separate evaluation of safe return to driving after TAA [2].

#### ChatGPT response analysis of question 6 (satisfactory response requiring minimal clarification)

Sports activity represents a significant part of social life, hence, after TAA, most patients inquire about postoperative expectations. It is observed that physical and sports activities tend to increase after TAA, but, actually, the rationale or preference regarding return to these activities are not standardized.

Regarding sport activities, after TAA, the most recommended ones are swimming, cycling, hiking and fitness/weights. In general, surgeons are comfortable with patients partaking in aerobic or low impact sports or activities after TAA [28] and high impact, cutting and jumping sports and activities are largely discouraged and seem to be allowed for only few and selected patients [18]. Young age, high body mass index and poor bone quality are negative factors for the return to sport activity, while low body mass index and patients with noninflammatory osteoarthritis have a 62% chance to play sports after surgery and in most cases can return to preoperative activity level. However, there are very few of studies dealing with sports participation in patients who undergo TAA, without strong evidence supporting the data [18].

#### ChatGPT response analysis of question 7 (satisfactory response requiring moderate clarification)

ChatGPT response is not complete. The existing literature does not offer insights into the extent of postoperative pain and its typicality during the initial phases. Additionally, it is imperative to differentiate between the transient postoperative discomfort that wanes with time and the persistent chronic pain that endures unresolved.

Immediately following TAA, oral NSAIDs, activity modification and immobilization with a lace‐up ankle brace or a CAM boot are employed to nonsurgically manage soft‐tissue inflammation, facilitating a gradual return to physical activity. In addition, to further reduce pain associated to synovitis or subtalar arthritis, physical therapy, such as stretching and strengthening and a local injection of anaesthetic and a corticosteroid can be used [9].

Following TAA, the persistence of symptoms may indicate deep infection or periprosthetic fractures (2%–4% of cases) and, even, the progressive worsening of pain over time may bring toward implant loosening and subsidence [38]. In this case, nonsurgical management plays a limited role in management of failed TAA with chronic pain. In this case surgical intervention is required.

Among the other causes that arise pain after TAA, it should be remembered bony or soft tissue impingement, periprosthetic osteolysis and cysts, adjacent joint arthritis and sinus tarsi impingement and nerve injury or compression [24].

#### ChatGPT response analysis of question 8 (satisfactory response requiring minimal clarification)

About this argument, ChatGPT gives a response that requires only minimal clarification because it proposes point by point the cases in which ankle fusion is better than TAA.

The primary surgical procedures in case of end‐stage ankle osteoarthritis are TAA and ankle fusion.

Patients undergoing ankle fusion are usually younger (<60 years) with greater comorbidity burden driven by hypertension, diabetes mellitus, obesity and tobacco use compared to TAA ones [14].

Regarding outcomes, the topic remains subject to ongoing study, with results in the literature not entirely consistent. Liu et al. observed that, following a 6‐month follow‐up peri‐od, patient satisfaction and reported outcomes post‐TAA are comparable to those following ankle fusion, despite TAA exhibiting a higher complication rate. By the 12‐month mark, no significant discrepancies in clinical scores were evident; however, TAA resulted in more frequent reoperations and complications [35]. In terms of complication rates and reoperations, ankle fusion displayed a higher overall complication rate, whereas TAA demonstrated a higher rate of revision surgeries [17]. A recent randomized controlled trial reinforced the notion that TAA and ankle fusion produce similar clinical scores and incidence of adverse events, although the nature of these events differs. Specifically, TAA is associated with a heightened incidence of wound healing complications and nerve injuries, while ankle fusion is correlated with a greater prevalence of thromboembolism and nonunion, which may manifest symptomatically in 7% of cases [22].

Therefore, a definitive conclusion regarding which surgical intervention is superior remains the topic of much research and the decision to proceed with TAA or ankle fusion for end‐stage ankle osteoarthritis should be made on an individual patient basis. In addition, patient goals and expectations are important to guide the decision between the two treatments.

#### ChatGPT response analysis of question 9 (satisfactory response requiring moderate clarification)

ChatGPT makes a list of what happens in the hospital, day to day, starting from the day of TAA, but it doesn't take into consideration the factors that contribute to the length of hospital stay after TAA.

The length of stay after TAA is generally between 1 and 3 days. This depends on some patient features and on surgeon volume: length of stay in hospital is significantly higher in female than male patients, and in patients with chronic kidney disease, obesity and diabetes. No differences are found between uncomplicated diabetic and nondiabetic patients, and between smoking and nonsmoking patients [5].

Surgeons with volume ≥90th percentile recorder a lower length of stay compared to other surgeons [13].

Additionally, it's worth noting that the postoperative rehabilitation protocol suggested by ChatGPT, which advocates for initiating physical therapy on the first postoperative day, lacks consensus in the literature, thereby raising questions about its validity.

#### ChatGPT response analysis of question 10 (satisfactory response requiring minimal clarification)

The response of ChatGPT is comprehensive and, as can be seen from the argumentation below, is in line with the current literature.

A range of 24°–30° combined motion (dorsiflexion and plantar flexion) is needed for normal walking, 37° is needed for ascending stairs and 55° is needed for descending stairs.

Factors that influence range of motion after TAA are divided into preoperative, intraoperative and postoperative ones. The preoperative factors are patient age, motivation, preoperative activity level, body habitus and disease type and severity. The intraoperative factors include restoration of the anatomical joint line, accuracy of tibial and talar cuts and component positioning. Finally, the postoperative factors are recovery and rehabilitation: patient ability, pain tolerance, motivation and the therapist are factors that influence the success of physiotherapy.

In addition, also muscle imbalance, adaptive gait patterns and scar tissue development can influence the postoperative ankle motion [10, 34].

As reported in Table 3, the mean score of the responses generated by ChatGPT using the Mika et al. response accuracy score was 2 (IRR = 0.9), suggesting that the overall responses were satisfactory requiring minimal clarification. Regarding DISCERN score, the mean was 51.1 (IRR = 1), which is considered good [21].

Considering the five readability scores, it was found that 13.3 years of education are needed to understand ChatGPT's responses. Specific readability scores can be found in the Table 4.

**Table 4:** Individual readability scores.

Readability formula	Grade level
Flesch–Kincaid Grade Level	12
Gunning Fog Index	18.3
Coleman–Liau Index	12
Simple measure of gobbledygook index	12
Automated readability index	12

## DISCUSSION

ChatGPT responses to the most asked questions by patients to clinicians regarding TAA resulted to be good and fair, often requiring minimal clarification. ChatGPT seemed to provide an adequate internet support for patients' understanding regarding TAA surgery success and postsurgical recovery. The accuracy of ChatGPT responses was proven and accepted by the foot and ankle surgeons and was supported by literature data. The results obtained in this study were consistent with other similar studies already published on different orthopaedic topics, and the responses were even better in terms of quality. This is probably because there were no paragraph limits when posing questions to ChatGPT, allowing it to provide comprehensive information without preselection.

The sole dissatisfying aspect of the responses concerned postoperative management of pain and the hospital stay, areas where consensus within the scientific community is lacking. AI failed to inform the patient that various postoperative protocols could be adopted, instead describing only one type, despite the current absence of evidence supporting its superiority over others.

This situation could potentially confuse patients if they seek treatment at an institute that applies a protocol different from the one suggested by ChatGPT. Fortunately, the consistent recommendation to seek guidance from an orthopaedic surgeon is crucial as it serves to delineate and constrain the role of AI in healthcare communication. As a matter of fact, the use of AI for critical patient information without thorough clinical assessment poses risks, both for the potential misinformation and for the incomplete guidance in complex cases.

The use of ChatGPT in the healthcare domain is increasingly growing, being utilized by healthcare professionals in research and by patients as an educational resource. The latter aspect constitutes a topic of great interest as it could provide a tool for all patients to gather information regarding their own conditions or proposed interventions. Moreover, the growing mistrust toward healthcare professionals and the persistent trend among patients to search for information on the internet may further encourage patients to turn to AI to confirm what has been told by their doctor or to seek answers. Consequently, various concerns have arisen within the medical community regarding the reliability of ChatGPT's responses to common questions posed by patients to healthcare professionals, which could also be directed to AI [6].

AI models have been already used in foot and ankle pathologies, for image analysis [1, 4, 5, 14], symptom, complication and surgery outcomes [20, 36].

In addition, several studies have been conducted to evaluate ChatGPT responses to common orthopaedic questions, but just few have been carried out on foot and ankle pathology. Parekh et al. raised some concerns about the accuracy of information provided by AI when comparing outcomes from various chatbots in response to inquiries about articles on common foot and ankle pathologies. Their investigation revealed insufficient accuracy across all chatbots, although ChatGPT emerged as the most reliable among them. However, it is important to note that the study used ChatGPT version 3, which had a knowledge cut‐off date of September 2021. Currently, the latest free version is ChatGPT 3.5, which is the version used in the present study with a cut‐off of January 2022 [26]. For this reason, the references included in the ‘Results’ section and employed for the discussion of the ChatGPT responses are dated before January 2022. The study by Anastasio et al. did not provide a clear conclusion regarding the quality of the responses given by ChatGPT. Rather, it concluded that there was a notable variability in the assessments made by the 11 observers, resulting in a very low IRR score [3].

On the contrary, studies conducted on other areas such as hip replacement and rotator cuff tendinopathy have shown good interreliability and found that most responses provided by ChatGPT were of good quality in terms of content, requiring minimal to moderate changes in most cases. The analysis on the readability of responses has shown that they were indeed understandable to a wide audience [21, 37].

The present study is the first to analyse ChatGPT responses to the most common questions asked by patients about TAA. The questions were developed by expert foot and ankle surgeons based on the questions that were very often asked to them by patients who underwent TAA. Consequently, the questions were very relevant and pertinent. In this study, different tools were used for the evaluation of ChatGPT responses, to have all‐round knowledge on the responses. Besides the two scores employed for the evaluation of satisfaction of ChatGPT responses, the readability of the answers was also calculated. With the use of a free readability checker, the ease with which the audience can read a written content was evaluated. The indices were in line with the other literature studies (value = 12), indicating that the responses had an advanced reading level, and the school level was college.

The limitation of this study primarily lies in posing questions to the chatbot only in English language. It would be interesting to assess whether the quality and readability of responses maintain this high level even in other languages to represent the patient's experience most accurately. Additionally, it would have been useful to evaluate whether the responses remain consistent or vary depending on whether the question is rephrased or paraphrased, reiterated or asked by different users, which could possibly be due to individual user metadata. AI tends to alter the wording of responses, but the meaning should remain unchanged. It is, therefore, plausible to hypothesize that accuracy scores would remain consistent, while readability scores might vary.

Moreover, this study aimed to analyze ChatGPT's responses to questions about TAA, which, despite the various models available, still present a fairly standardized protocol and a good level of scientific agreement. A starting point for further studies would be to evaluate how ChatGPT responds to questions on topics that are still highly debated even within specialist circles, to ascertain how and if AI will present diverging opinions. Furthermore, we posed the questions only once, and it is uncertain whether the quality of the responses would remain consistent if the questions were repeated.

Another limitation is that the two scores (Mika et al. and DISCERN), employed for the evaluation of the ChatGPT responses, were subjective. However, these are the most employed score in this ambit, and they were used by two independent authors, in order to reduce any bias. In this regard, it was observed that the IRR values were acceptable (0.9 and 1) [21].

## CONCLUSIONS

ChatGPT currently offers valuable support for patients seeking information about TAA surgery. The responses provided are largely comprehensive, typically necessitating only minimal to moderate clarifications. Moreover, the consistent recommendation to consult an orthopaedic surgeon appropriately underscores the role of AI in healthcare.

As a matter of fact, the use of AI for critical patient information without thorough clinical assessment poses risks, including the potential for misinformation or incomplete guidance in complex cases. As AI tools like ChatGPT rely heavily on the available literature and lack clinical judgement, their role should only complement, rather than replace, direct patient–surgeon communication.

However, ChatGPT demonstrates potential as a tool for responding to common patient questions related to TAA, offering clear and mostly accurate information.

While its current performance is based on the available literature, ongoing advancements in AI may further enhance its utility in healthcare communication. Further studies are required to evaluate its role more precisely in patient information and clinical settings.

## AUTHOR CONTRIBUTIONS

All authors contributed to the study conception and design. Material preparation, data collection and analysis were performed by Francesca Veronesi, Elena Artioli and Antonio Mazzotti. The first draft of the manuscript was written by Francesca Veronesi and Elena Artioli. Formal analysis and data curation were performed by Silvia Brogini and Simone Ottavio Zielli. Supervision and editing of final manuscript were performed by Gianluca Giavaresi and Cesare Faldini. All authors commented on previous versions of the manuscript. All authors read and approved the final manuscript.

## CONFLICT OF INTEREST STATEMENT

The authors declare no conflicts of interest.

## ETHICS STATEMENT

The authors have nothing to report.

## Data Availability

The original contributions presented in the study are included in the article, further inquiries can be directed to the corresponding author.
